# Machine-learning algorithms based on personalized pathways for a novel predictive model for the diagnosis of hepatocellular carcinoma

**DOI:** 10.1186/s12859-022-04805-9

**Published:** 2022-06-23

**Authors:** Binglin Cheng, Peitao Zhou, Yuhan Chen

**Affiliations:** 1 Department of Radiation Oncology, Nanfang Hospital, Southern Medical University, 1838 Guangzhou Avenue North, Baiyun District, Guangzhou, 510515 Guangdong Province China; 2 The First School of Clinical Medicine, Southern Medical University, Guangzhou, Guangdong Province China

**Keywords:** Hepatocellular carcinoma, Diagnostic model, Machine learning, Pathway, Prognosis

## Abstract

**Background:**

At present, the diagnostic ability of hepatocellular carcinoma (HCC) based on serum alpha-fetoprotein level is limited. Finding markers that can effectively distinguish cancer and non-cancerous tissues is important for improving the diagnostic efficiency of HCC.

**Results:**

In this study, we developed a predictive model for HCC diagnosis using personalized biological pathways combined with a machine learning algorithm based on regularized regression and carry out relevant examinations. In two training sets, the overall cross-study-validated area under the receiver operating characteristic curve (AUROC), the area under the precision-recall curve and the Brier score of the diagnostic model were 0.987 [95%confidence interval (CI): 0.979–0.996], 0.981 and 0.091, respectively. Besides, the model showed good transferability in external validation set. In TCGA-LIHC cohort, the AUROC, AURPC and Brier score were 0.992 (95%CI: 0.985–0.998), 0.967 and 0.112, respectively. The diagnostic model has accomplished very impressive performance in distinguishing HCC from non-cancerous liver tissues. Moreover, we further analyzed the extracted biological pathways to explore molecular features and prognostic factors. The risk score generated from a 12-gene signature extracted from the characteristic pathways was correlated with some immune related pathways and served as an independent prognostic factor for HCC.

**Conclusion:**

We used personalized biological pathways analysis and machine learning algorithm to construct a highly accurate HCC diagnostic model. The excellent interpretable performance and good transferability of this model enables it with great potential for personalized medicine, which can assist clinicians in diagnosis for HCC patients.

**Supplementary Information:**

The online version contains supplementary material available at 10.1186/s12859-022-04805-9.

## Introduction

Hepatocellular carcinoma (HCC), a quite common primary liver malignant tumor, is the major reason of cancer-associated death all over the world [1]. Timely and accurate diagnosis is essential for improving the therapeutic efficacy of HCC. In addition to pathological diagnosis, HCC is usually diagnosed by serum alpha-fetoprotein (AFP) levels and imaging results at present [2]. However, it is reported that the sensitivity of AFP for HCC diagnosis is about 60% even tested by a low-level cutoff [3]. And because AFP levels also increase in other system disorders or benign liver diseases, the specificity of AFP to diagnose HCC is still insufficient [4]. Therefore, a great deal of new biomarker have been found to improve the diagnosis of HCC. But these biomarkers have not entered the stage of clinical trials yet, and many of them are still inadequate in terms of sensitivity such as Glypican-3 and Golgi protein-73 [5]. Thus, it’s of vital importance to ascertain more accurate predictors for diagnosis of HCC.

In recent years, the role of machine learning algorithm in supporting HCC medical work can not be ignored. In terms of prognosis, Santos et al. utilized a cluster-based oversampling method based on the K-means clustering and SMOTE algorithm to build a model for predicting the 1-year survival of HCC patients, and the model achieved the best classification efficiency of 75.19% [6]; Chicco et al. used random forest algorithm for survival prediction and pointed out that alkaline phosphatase, AFP and hemoglobin levels were the most predictive survival factors for HCC, which brought great help to practical medical application [7]; Ksiazek et al. constructed two models to predict the survival of HCC patients with different approaches, one of which fused genetic algorithms and logistic regression [8], and the other included neighborhood components analysis, genetic algorithm and support vector machine classifier [9]. Their accuracy was all over 94%, which meant they can be applied to the evaluation of HCC mortality in the future. As for diagnosis, previous studies have combined deep learning classifier with contrast-enhanced magnetic resonance imaging [10], computed-tomography images [11] and ultrasound images [12] respectively to build HCC diagnosis models, which have better performance than most doctors only diagnosing by images; More importantly, based on genomics, machine learning algorithms were used for HCC diagnosis, such as three models separately based on 3 genes, 5 CpG sites and 5 RNA transcripts constructed by Kaur et al. These models all utilized a variety of machine learning algorithms such as logistic regression, support vector machine, random forest and neural network, and all achieved accuracy of more than 95% [13, 14]. However, with the rapid expansion of data, the efficiency of machine learning model has more opportunities to be further improved. At the same time, previous algorithms are more and more difficult to adapt to more and more complex datasets, so the demand for new machine learning algorithms will not stop.

High-throughput technologies can provide a huge number of features and high-dimensional data for tumor [15]. Then omics research can take what we receive as a whole and extract meaningful information from it [16]. Nevertheless, because of small sample sizes of high-throughput technology, the problem of High-Dimension Low Sample Size (HDLSS) arises, which brings great challenges to our studies, such as the so-called “curse of dimension” [15]. Besides that, on account of the intrinsic multicollinearity among predictors, traditional methods generally fail to select predictors from omics data. In addition, many nonlinear approaches for variable selection may overfit the data. Thus linear models have proved to be more recommended at present [17].

The least absolute shrinkage and selection operator (Lasso) and ridge regression are common regularized regressions with a capability to provide an approach to fit generalized linear models whose coefficients are limited [18]. Lasso, a frequently-used penalized regression method, achieves variable selection by imposing the L1 penalty to traditional least squares and offering sparsity inducing estimation. However, if multicollinearity occurs among predictors, lasso tends to select only one of them. When the number of predictors is greater than the number of observations (n), at most n predictors can be selected in the model. Therefore, some important variables will be lost, resulting in the model performance failing to meet expectations [19]. Ridge regression reduces the loss of function which contains the sum of squared regression residuals to the minimum through the L2 norm of the coefficients [20]. Ridge will not have lasso’s problems, but the results of ridge regression are not concise relatively. Partly because the elastic-net penalty combines L1 and L2 norm penalties, elastic-net regression overcomes the defects of lasso and ridge. Previous studies have shown that elastic-net performs better than the first two methods in dealing with high-dimensional data [21], which makes elastic-net a preferred choice when facing HDLSS.

Nowadays, the amount of omics data is growing exponentially, presenting both opportunities and challenges for many researchers. In the age of biomedical big data, the major trouble of research is how to analyze data of different types and sources to obtain novel viewpoints. Especially when dealing with large-scale datasets, varieties of data types and complicated designs, omics data analysis lacks universal systematic methods, and its performance is also far from ideal [22]. Nevertheless, it was demonstrated that pathway activity scoring approaches and prediction methods can improve the robustness, accuracy and biological interpretability of models through dimension reduction approaches [23]. Pathifier algorithm is an effective method for pathway-related research, which has been successfully applied in diagnostic model development of papillary thyroid carcinomas [24]. Pathifier can convert gene-level information into pathway-level after dimension reduction and generate a pathway deregulation scores (PDS) for each sample, which could successfully reflect the level of pathway-related dysregulation, so as to realize personalized pathway-level analysis [25].

**Fig. 1:**
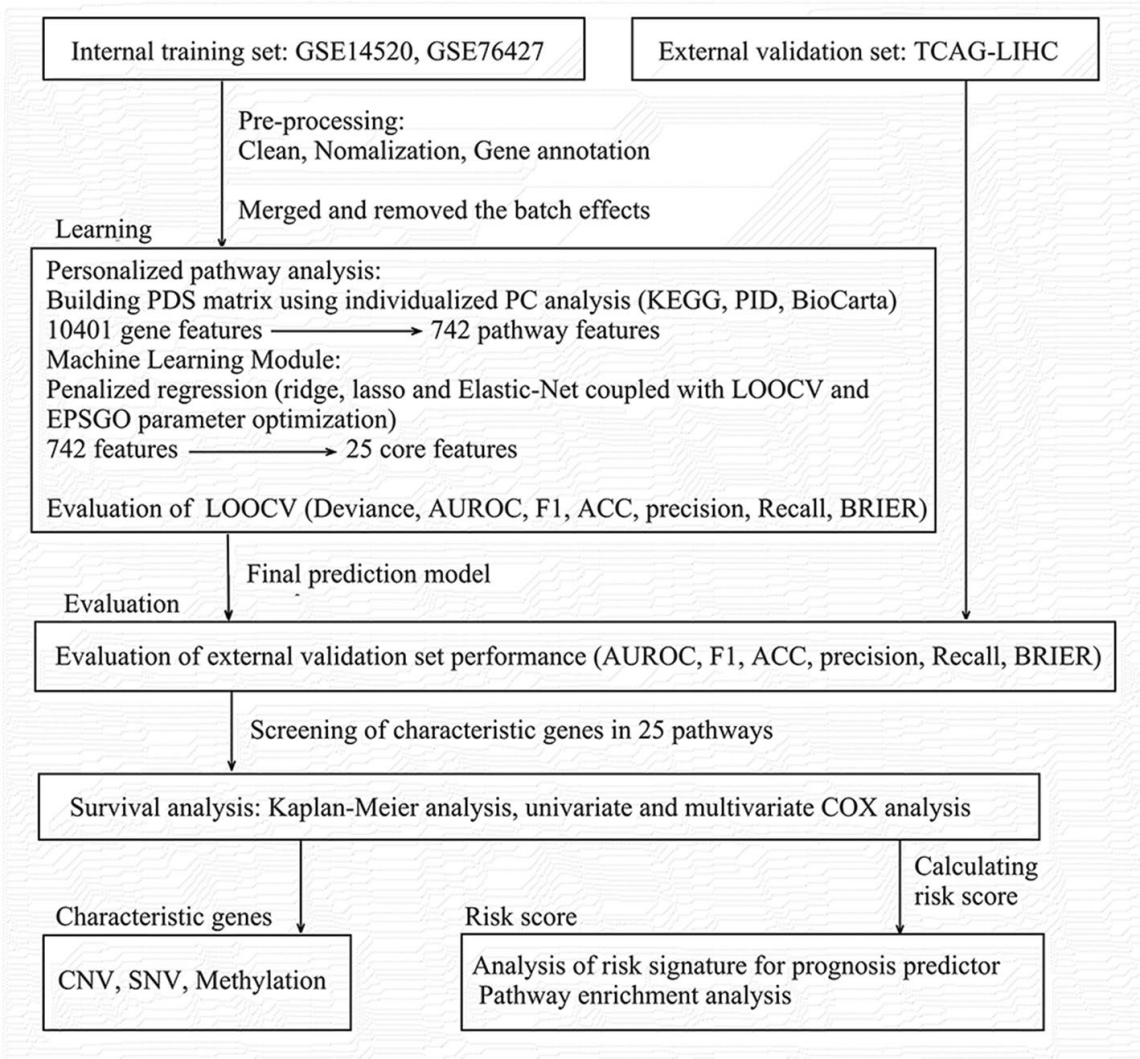
The flowchart of study design

In this study, we used personalized biological pathways analysis and machine learning algorithm consisting of regularized regression to construct a highly accurate and multi-study-derived HCC diagnostic model, which showed very impressive performance in the validation set. This was a diagnostic prediction model based on gene pathway information, which might be used as a supplement to clinical pathological diagnosis and a reference for follow-up gene-related therapy of HCC patients. And its high accuracy and transferability makes it play a great role in personalized medicine, making accurate diagnosis for HCC patients to enhance clinicians’ decision. Moreover, we also found new changes in pathways and prognostic characteristics of HCC, which could provide directions for subsequent study. The study design was shown in Fig. 1.

## Results

### Construction of a PDS matrix to transform gene information into pathway features

First of all, two training sets were merged with the empirical Bayes algorithm (Additional file 1: Fig. S1). Pathifier algorithm was utilized to transform gene expression level data from the merged training sets into a pathway level matrix in training sets. Pathifier took advantage of an algorithm by Hastie and Stuetzle to get a principal curve that was nonparametric and nonlinear generalization of the first several principal components with regard to dimension reduction [26]. In this way, a one-dimensional principal curve was yielded by analyzing data points from a cloud in the high dimensional space, and every sample’s PDS was calculated by the distance from the starting point of the principal curve (the centroid of the control samples) to the target point of the personalized pathway projection. Then for every sample, we could yield a compact pathway representation ultimately.

**Fig. 2:**
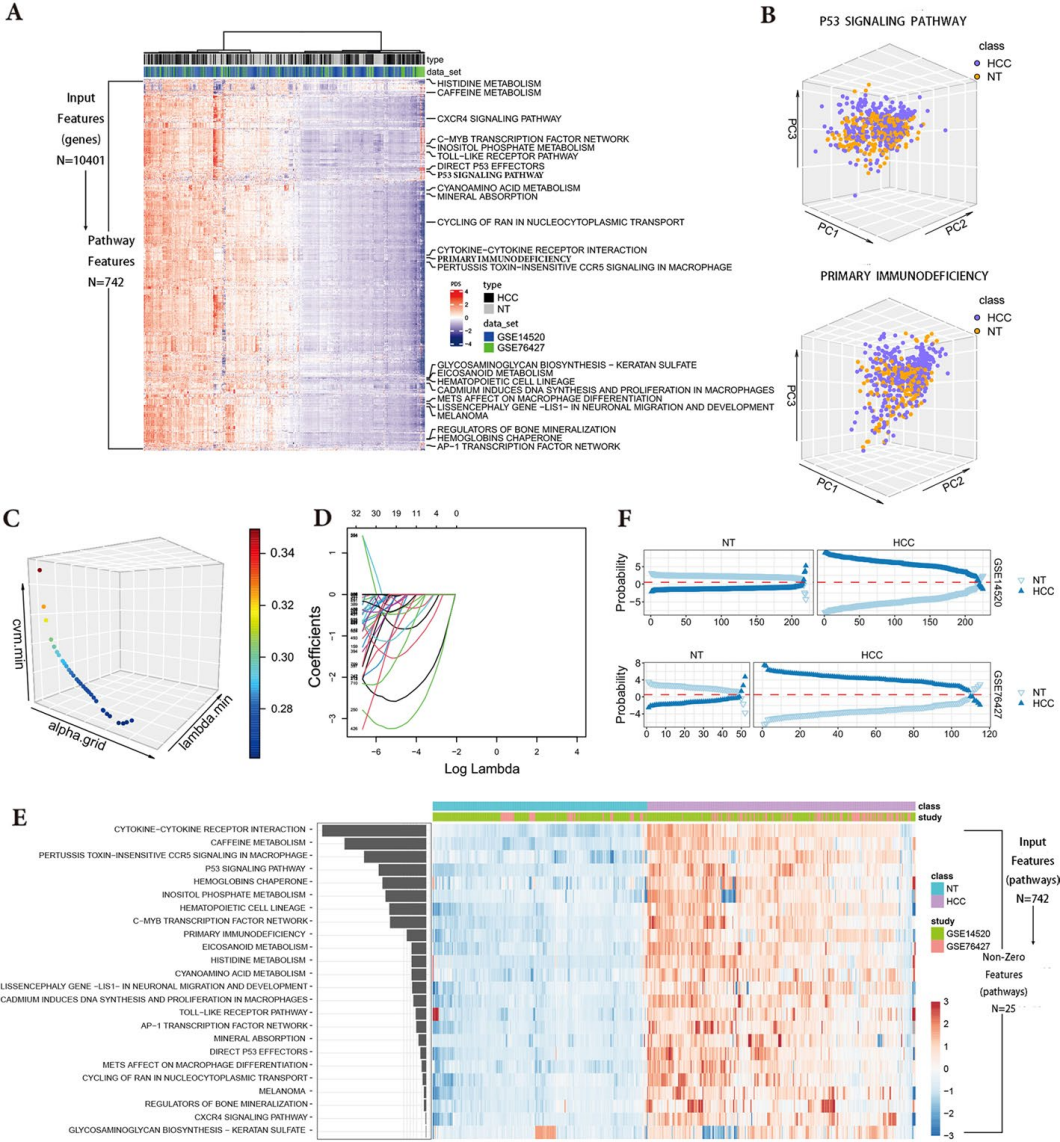
Construction of diagnostic model. **A**Construction of a PDS matrix for the training cohort. **B** Principal component analysis of selected pathways. **C** Elastic-net penalized regression models with EPSGO were performed to obtain the optimal hyperparameters α and λ (α = 0.85372, λ = 0.004230762, deviance = 0.03503). Among them, α represents the balance between lasso and ridge penalties. A closer α to the arrow direction indicates that the model was more like lasso regression, otherwise it was more like ridge regression. And the total amount of penalization was controlled by λ. As for CVM (cross-study validation method), the deviance of cross-study validation, was used to measure the effectiveness of modeling. Thus the lowest point of the curve with the minimum deviance was the final EPSGO solution. **D** The selection of non-zero coefficients regard to hyperparameter λ. Each curve corresponds to a predictor. The numbers above the box mean the numbers of non-zero coefficients with their corresponding log(λ). And the Y-axis was each predictor’s coefficient, gradually approaching 0 as λ increases. **E** Heatmap of 24 non-zero coefficient pathways. **F** Cross-study validation for estimated probabilities of each sample. PC, principal component; EPSGO, efficient parameter selection via global optimization; NT, non-tumoral

We merged two training datasets and one external validation dataset according to genes after annotation. Finally, 10,401 shared genes were obtained and used as input features. Then we conducted each pathway’s principal component analysis (Fig. 2B) and constructed the pathway signatures, namely a PDS matrix with 742 rows (Fig. 2A). According to the method of regularized regression, we used the PDS matrix to established a diagnostic model for HCC. Elastic-net regularization was an awesome method for statistical modeling based on a combination of the ridge and lasso regression, in which two hyperparameters (α and λ) needed be fine-tuned to obtain a suitable elastic-net penalty function. The trade-off between the ridge and lasso penalties was dominated by α, while the total amount of penalization was controlled by λ [27]. Due to the high arbitrariness of the generally used fixed grid search approaches, an algorithm called Efficient Parameter Selection via Global Optimization (EPSGO) [28] was chosen to seek the best value of α and λ with minimum binomial deviance (Fig. 2C). When the binomial deviance given from the regularization parameter became the lowest, EPSGO-tuned elastic-net managed to generate a group of predictors containing 24 pathways with non-zero dysregulation coefficients (Fig. 2D–E). And the model performed well in cross-study validation eventually (Fig. 2F).

### Multi-index estimation of the diagnostic model’s accuracy

**Fig. 3:**
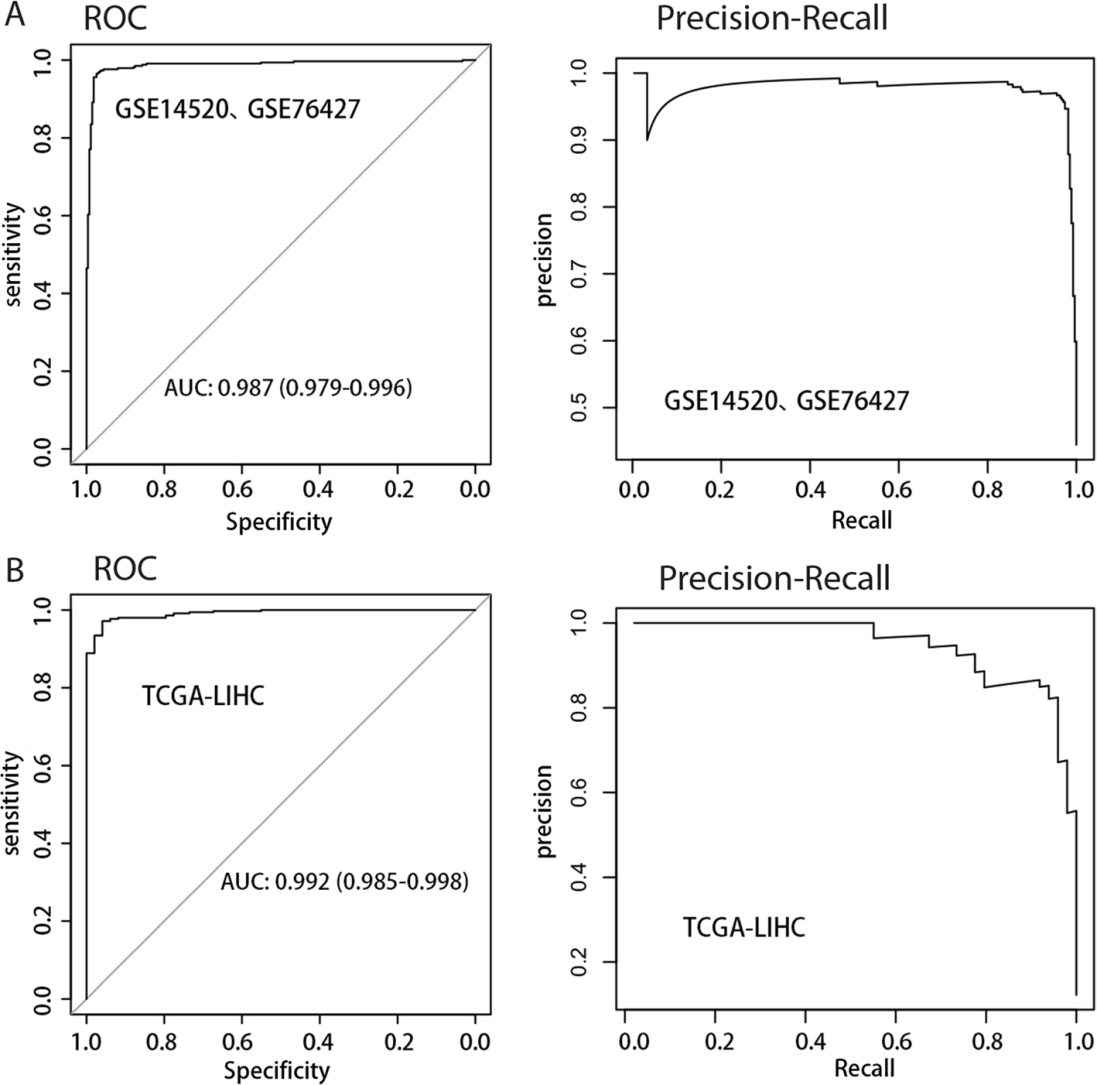
Internal **A** and external **B** validation of model performance for distinguishing HCC and non-tumor samples by using receiver operating characteristic (ROC) and precision-recall curve

The confusion matrix of training set and validation set were shown in Tables 1 and 2 respectively. The area under the receiver operating characteristic curve (AUROC) of training cohort was 0.987 (95%CI: 0.979–0.996) and the area under the precision-recall curve (AUPRC) was 0.981. The Brier score was 0.091. The sensitivity, precision and Matthews correlation coefficient (MCC) were 0.965, 0.976 and 0.934 respectively (Table 3, Fig. 3A). As for external validation cohort TCGA-LIHC, its AUROC, AURPC and Brier score were 0.992 (95%CI: 0.985–0.998), 0.967 and 0.112 respectively and with a sensitivity of 0.849, a precision of 1 and a MCC of 0.639 (Fig. 3B, Table 3), which was indeed encouraging and confirmed that our diagnostic model had great performance. Although some high-confidence HCC models have been developed, the inconsistent research methods limit their directly comparison. If assessed from the AUROC, the AUROC values of our model were higher than those of most diagnostic models that have been reported so far (Additional file 8: Table S1).

**Table 1:** The confusion matrix of training set

GSE14520 and GSE76427		True condition	
		HCC	Non-HCC liver tissues
Predicted condition	HCC	328	8
	Non-HCC liver tissues	12	264

**Table 2:** The confusion matrix of validation set

TCGA-LIHC		True condition	
		HCC	Non-HCC liver tissues
Predicted condition	HCC	298	0
	Non-HCC liver tissues	53	49

**Table 3:** Performance evaluation of training and external validation sets

	Overall cross-study validation	External cohort validation
	GSE14520, GSE76427	TCGA-LIHC
AUPRC	0.981	0.967
AUROC	0.987(0.979–0.996)	0.992(0.985–0.998)
Brier score	0.091	0.112
ACC	0.967	0.868
Precision	0.976	1
Sensitivity (recall)	0.965	0.849
F1	0.97	0.918
MCC	0.934	0.639

### Analysis of hub gene network in pathway features

Based on regression and path information, our machine learning algorithms provide more biologically explainable results than other so-called “black box” machine learning algorithms. The algorithm generated 24 non-zero coefficient HCC related pathways with the largest amount of information in the elastic network model. Among these pathways, 824 characteristic genes were identified. Based on these genes, we constructed protein protein interaction (PPI) network using STRING database (Additional file 2: Fig. S2A) and finally 753 characteristic genes were extracted from the network. We further conducted pathway enrichment analysis through g:Profiler database [29]. The top ten pathways enriched by KEGG were Cytokine-cytokine receptor interaction, Pathways in cancer, JAK-STAT signaling pathway, PI3K-Akt signaling pathway, Melanoma, Hematopoietic cell lineage, Viral protein interaction with cytokine and cytokine receptor, Hepatitis B, Human cytomegalovirus infection, and Measles. The detailed enrichment results referred to the supplemental materials (Additional file 9: Table S2). Then a hub network was built based on the top 20 genes ranked by degree via Cytohubba (Additional file 2: Fig. S2B).

### Prognostic significance of genes involved in model-related pathways

**Fig. 4:**
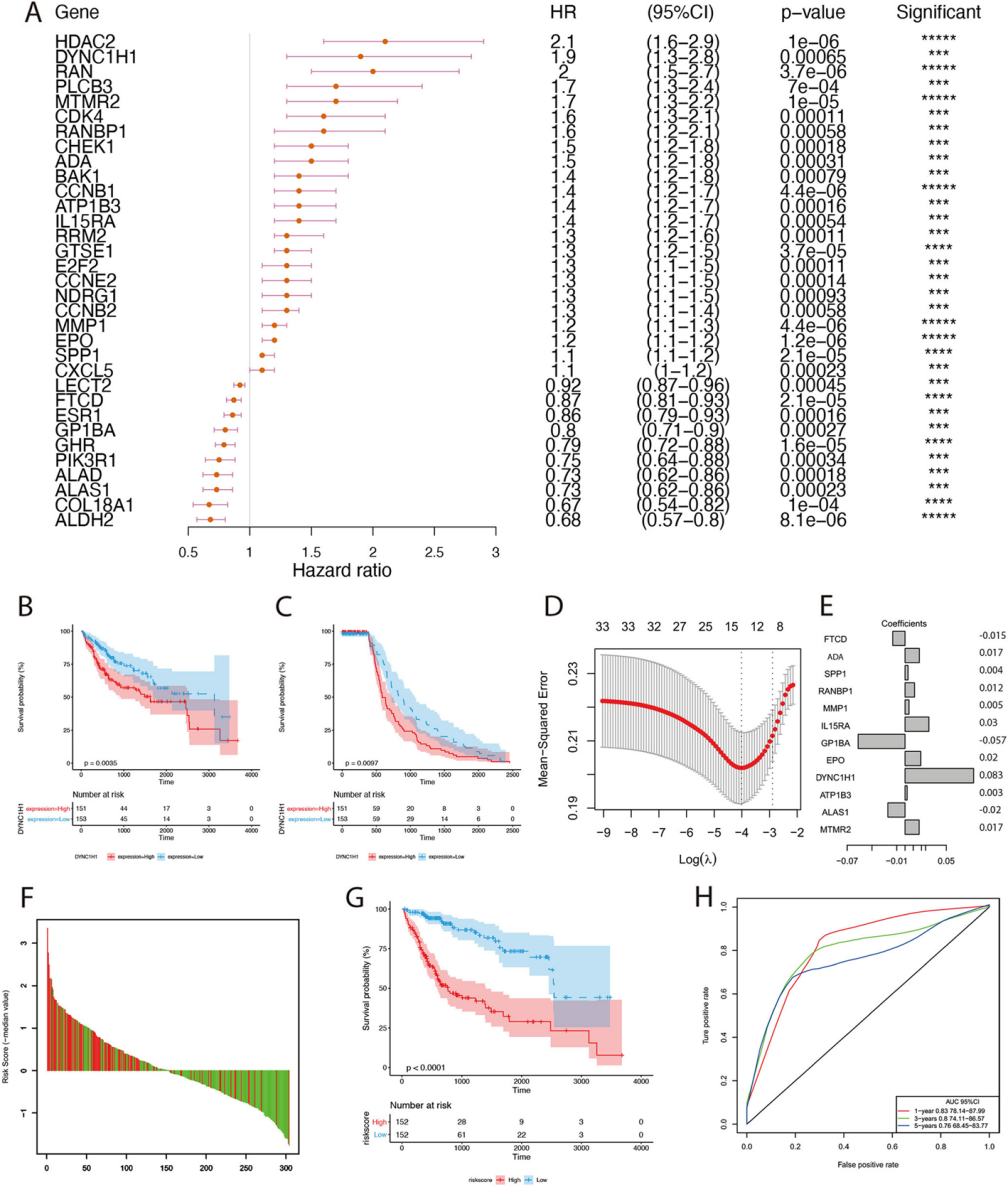
Prognostic values of characteristic genes. **A** HR statistics of 33 prognostic-related genes. **B** The Kaplan–Meier survival curve of OS for DYNC1H1. **C** The Kaplan–Meier survival curve of RFS for DYNC1H1. **D** Lambda distribution of lasso regression. The left line indicated the optimal values (λ = 0.01988275). **E** lasso regression coefficient of 12 genes. **F** Analysis of the correlation between the risk score and survival status. **G** The Kaplan–Meier survival curve of 12-gene signature in TCGA cohort. **H** The AUC curve of 12-gene signature in TCGA cohort. *: *P* ≤ 0.05, **: *P* ≤ 0.01, ***: *P* ≤ 0.001, ****: *P* ≤ 0.0001, *****: *P* ≤ 0.00001

Based on 824 characteristic genes mentioned above, we performed univariate Cox survival analysis on TCGA cohort to evaluate the prognostic values of these genes and eventually we found 33 genes correlated with overall survival (OS) (*P* < 0.001, C-index ≥ 0.6) (Fig. 4A, Additional file 10: Table S3). Among them, PIK3R1 was also identified in the hub network mentioned above (Additional file 2: Fig. S2B). Through Cox survival estimation and Kaplan–Meier survival analysis, HDAC2 was the most significant negative prognostic factor for OS, while ALDH2 became the most significant positive prognostic factor. Moreover, only DYNC1H1 high expression exhibited both worse OS (Fig. 4B) and relapse-free survival (RFS) (Fig. 4C) with significant difference by Kaplan–Meier survival analysis of 33 genes (Additional file 3: Fig. S3).

In order to reduce the number of those genes with prognostic value, we performed lasso regression analysis on above-mentioned 33 genes and obtained 12 notable characteristic genes (Fig. 4D–E). We further investigated whether these 12 characteristic genes were differentially expressed between non-cancer tissues and cancer tissues of different stages (stage I-IV). The results showed that ADA, ATP1B3, DYNC1H1 and FTCD were differently expressed in non-cancer vs. cancer tissues of different stages, in cancer tissues of stage I vs. stage II or stage I vs. stage III (Additional file 4: Fig. S4). This will help determine the markers for early stages of tumorigenesis and cancer progression. Based on these 12 genes, we carried out multivariate Cox regression analysis in TCGA cohort (Additional file 11: Table S4) to construct the risk signature and took the sum of the product of each gene’s coefficient and each gene’s expression as the risk score. The formula was as follows: risk score = (0.008107*ADA expression) + (0.035155*ALAS1) + (− 0.032727*ATP1B3) + (0.217810*DYNC1H1) + (0.101323*EPO) + (− 0.280598*GP1BA) + (0.160445*IL15RA) + (0.023708*MMP1) + (0.098353*RANBP1) + (0.055255*SPP1) + (0.301356*MTMR2) + (− 0.083692*FTCD). The survival analysis based on risk score showed that in the TCGA cohort, there was a remarkable survival difference between high- and low-risk groups which were divided by the median of risk scores (Fig. 4F, G) The AUCs of 1-year, 3-year and 5-year survival analysis were all greater than 0.76 (Fig. 4H). Moreover, the multivariate Cox regression analysis pointed out that the risk score could serve as an independent prognostic factor (Additional file 12: Table S5).

### Subgroup analysis of the risk signature for prognosis prediction

For evaluating efficiency and stability of the risk signature, we extracted clinical characteristics from the TCGA cohort, including age, gender, clinical stage, stage_T, stage_N, stage_M, grade and recurrence. In view of above information, we compared survival difference of the 12-gene risk signature in the subgroups, and found that this risk signature could also predict the significant prognostic differences in these subgroups (Additional file 5: Fig. S5), which indicated that the efficiency and stability of prognosis prediction of this signature were pretty good.

### Correlation between the risk signature and immune related pathways

We collected 19 immune-related pathways, and analyzed the relationship between the GSVA enrichment scores and the risk score of 12-gene risk signature. Then we found that risk score was positively correlated with cell cycle, DNA replication and homologous recombination, but negatively related to CD8 + T. These results indicated that high risk score was associated with enhanced cell proliferation and suppression of immune response (Additional file 6: Fig. S6).

### Mutation estimation of characteristic genes

**Fig. 5:**
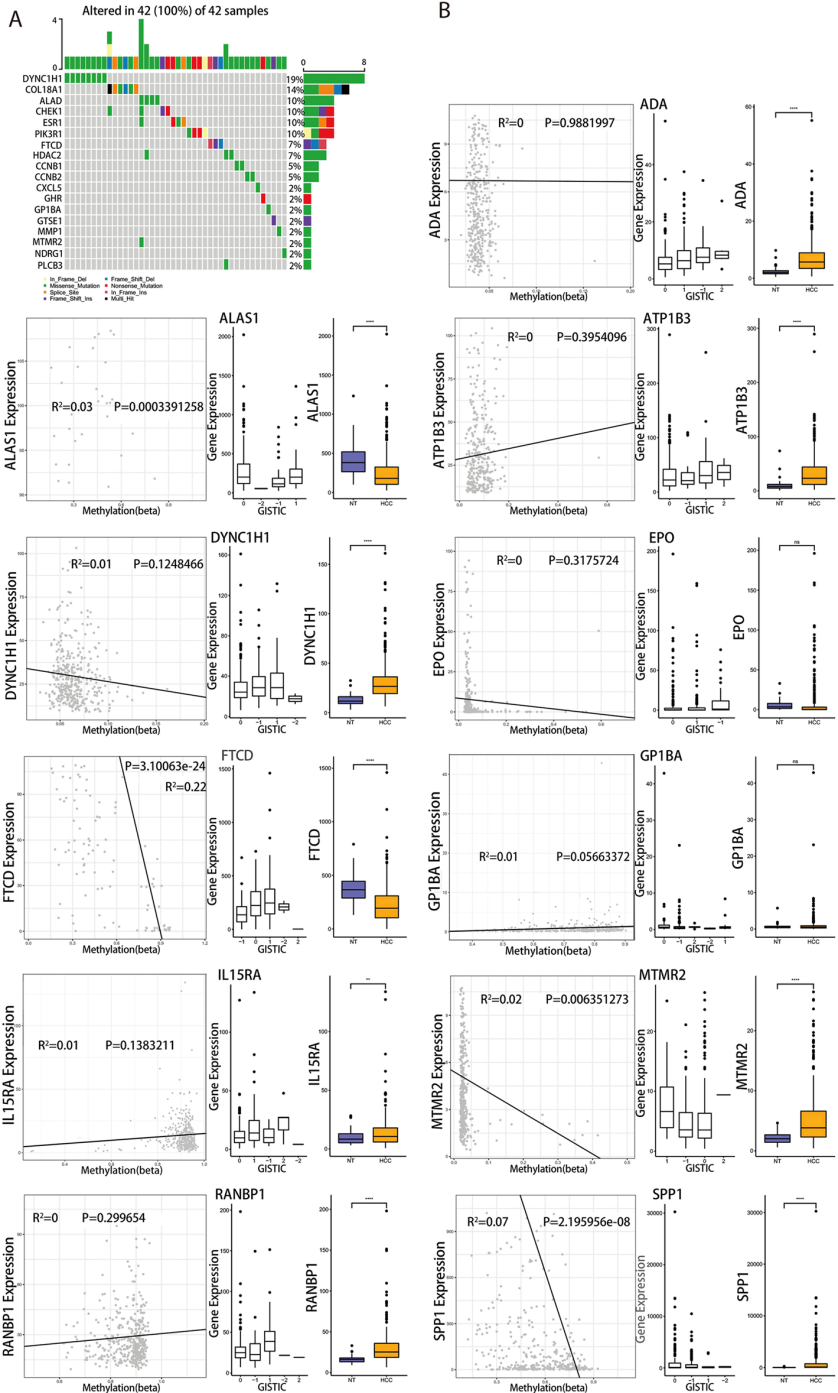
Mutation assessment of characteristic genes. **A** Single nucleotide variations of 33 genes. **B** Analysis of correlation between the expression level of 11 genes and methylation and copy number variation level. *: *P* ≤ 0.05, **: *P* ≤ 0.01, ***: *P* ≤ 0.001, ****: *P* ≤ 0.0001

Next, we analyzed the mutations of 33 genes related to prognosis in univariate Cox analysis. As shown in Fig. 5A, single nucleotide variations (SNVs) were detected in 18 of these 33 genes and these mutated genes were distributed in 42 samples. Especially, DYNC1H1 harbored the largest number of SNVs in TCGA-LIHC cohort. But among the 12 characteristic genes, only 5 with SNVs were detected in 14 samples (Additional file 6: Fig. S6), which indicated that the functional alteration caused by SNV in these genes may not be an important pathogenic factor in HCC. Therefore, we then focused on the relationship between the expression of 12 characteristic genes and the levels of copy number variation (CNV) and methylation (MMP1 was excluded because of no methylation data). Except for EPO and GP1BA, the other 9 genes showed significant differences in their expressions between HCC and non-tumor tissues in TCGA cohort (Fig. 5B, right panel). We further validated the expression of 12 characteristic genes in HCCDB database (Additional file 7: Fig. S7). The expression of most genes from TCGA datasets were consistent with the results from some other datasets. For instance, DYNC1H1 and SPP1 were consensus up-regulated in 4 and 7 datasets, respectively. While ALAS1 and FTCD were consensus down-regulated in 5 and 10 datasets, respectively. It indicated that the expressions of these genes are relatively stable in HCC. In addition, the expressions of these genes in HCC samples were also varied with CNV and mostly positively correlated with copy number. (Fig. 5B, middle panel). In order to simplify the analysis and ensure the comparability between different genes, the correlation of gene methylation levels and expression levels was analyzed using linear model through pearson correlation analysis. The results showed that the expressions of DYNC1H1, EPO, FTCD, MTMR2 and SPP1 were negatively correlated with methylation levels while most others showed the opposite patterns (Fig. 5B, left panel).

## Discussion

With the rapid growth of genetic information in recent years, machine learning has turned into an extremely important research tool because it can find complicated patterns in high-dimensional data to achieve various purposes [30]. However, because of the inner complexity, it is still very challenging in interpreting machine learning model, which is the so-called ‘black box’ [31]. Black box models have caused obstacles to the follow-up research and application. In order to solve this problem, the surrogate model strategy was utilized to increase the interpretability [32]. But owing to very complex logic principles behind black box models, the traditional surrogate model, such as decision trees, unable to learn the whole [31]. Therefore, for the sake of conquering these shortcomings, we converted gene expression levels of two training sets into pathway expression levels to reduce the dimension. Then we established the HCC prediction model through regularized regression and worked out 24 non-zero pathway predictors. The excellent interpretable performance of this model enables it with great potential for personalized medicine.

Despite the AUROC of 0.987 in our training sets has indicated the outstanding performance of this model, we have also used other evaluation approaches for further verification. Compared with ROC, PRC is more sensitive to imbalance and can better reflect the classification performance when there exists large proportion of difference between positive and negative samples [33]. As expected, the AUPRC of 0.981 in training sets demonstrates that our model has strong robustness. Due to discrimination and calibration, the Brier score is usually used to measure the accuracy of probability prediction [34]. The Brier score of 0.091 was also obtained to reflect the high accuracy of our model. Compared with accuracy and F1 score, the MCC is more informative and reliable [35]. The MCC of 0.934 makes the great performance of our model more realistic. Besides, the excellent statistical metrics of the model in training sets achieved a good reproduction in the verification set with an AUROC of 0.992, an AUPRC of 0.967, a Brier score of 0.112 and a MCC of 0.639, which illustrated the good transferability of the model. Compared with most previous reports (Additional file 8: Table S1), the diagnostic model of this study could achieve better diagnostic performance in both the training and the validation groups, suggesting that it has good application potential. In previous studies, Kaur et al. used a variety of traditional machine-learning algorithms to construct classifiers for HCC and normal tissues. If assessed from the AUROC, the AUROC values of their three models based on 3 genes [13], 5 CpG sites and 5 RNA transcripts [14] were 0.96–0.99, 0.94–99 and 0.93–97 respectively, showing slightly worse performance than ours. In addition, it has been reported that pathway based analysis can provide more insights into the complex biological mechanisms of diseases than genomics based analysis [25]. So biological interpretation is an important characteristic of our model, which is also what previous models lack. The clinical stage is one of the important prognostic factors for HCC. Interestingly, our diagnostic model could also obtain the AUC of 0.932 (95%CI: 0.900–0.963) for the distinguishment of stage I-II from stage III-IV. However, it may not be the optimal model because our diagnostic model was designed for distinguishing HCC from non-cancer cases but not for distinguishing the different stages of HCC. We will try to develop the diagnostic model for different stages in future.

Based on the 824 characteristic genes in 24 pathway predictors, 33 were determined as significant prognostic factors. Previous studies have demonstrated the important role of some characteristic genes, such as HDAC2, RAN and PLCB3, involving in the progression of HCC [36–38]. which may aid in the development of diagnostic and prognostic biomarkers for HCC. Among these genes, DYNC1H1 was the only prognostic factor both related to OS and RFS and a gene with the largest number of single nucleotide mutations. A recent study reported that mutant DYNC1H1 may serve as a biomarker for the therapy of microtubule inhibitors in gastric cancer with high immune activity [39]. However, there is still a lack of study on the relationship between DYNC1H1 mutation and HCC, which is also the direction of our future research.

Utilizing lasso and multivariate Cox regression analysis, we screened 12 characteristic genes to construct a risk signature. High risk score generated by the risk signature was positively correlated with the poor survival outcome of HCC patients. In addition, the risk stratification revealed significant prognostic differences within the subgroups including age, gender, grade, stage and recurrence, which suggested that the risk signature has a great potential in clinical use. Moreover, we also found that risk score is positively correlated with cell cycle, DNA replication and homologous recombination, all of which have been confirmed to have relation to the progression of HCC [40–42]. Meanwhile, risk score was negatively related to CD8 + T. And the exhaustion of CD8 + T cell has been verified to be associated with HCC progression [43]. These findings indicated that high risk score was associated with enhanced cell proliferation and suppression of immune response, which partly accounted for the poor prognosis in the high-risk group.

There were several limitations in this study. First, the data included in this study were related to very diverse HCC subtypes, and only the gene expression data in cancer and non-cancer tissues were used for diagnostic model development. Due to the limitations of the original data, we cannot evaluate the normal purity of non-cancerous tissue, the condition of pre-malignant and the early malignant transformation such as the carcinoma in situ. Therefore, the constructed diagnostic model in this study can only be used to distinguish cancer from non-cancer, but not distinguish very early events from purely normal cases. Second, one of the current difficulties for clinical application is the lack of highly sensitive and accurate gene signature. Although our diagnostic model performed well on datasets with different sample sizes from different sequencing platforms, it still needs to be validated in some other cohorts to evaluate its clinical significance for HCC. Third, some key genes related to prognosis were screened out through lasso regression. However, the use of lasso regression cannot consider the biological information of genes, thus some genetic information will inevitably be lost. This is the limitation of the analytical method used in this study.

## Conclusions

In conclusion, this study established the HCC diagnostic model through personalized biological pathways analysis and machine learning algorithm. This model is not only capable of achieving highly accurate diagnosis of HCC, but also has a high degree of interpretability for HCC patients’ pathological results because of its personal pathway information, which provides substantial help for clinicians to determine each HCC patient’s diagnosis and treatments. We also found out some important characteristic genes related to prognosis, gene mutation as well as immune-related pathways, which is of significance for the understanding of HCC. Although the HCC diagnostic model harbored good performance, it still needs more verification sets to be continuously improved.

## Methods

### Data collection

The microarray data of GSE14520 [44] and GSE76427 [45] were downloaded by R package “GEOquery”. The transcriptome data of TCGA-LIHC were obtained from HCCDB [46], whereas the gene mutation data, methylation data, copy-number alteration data and corresponding clinical information of TCGA-LIHC were retrieved from UCSC XENA [47]. Criteria for study inclusion were: (1) The cases were diagnosed as HCC or non-HCC liver tissues. (2) HCC caused by different types of etiologies was acceptable. (3) The cases had complete expression data. (4) For clinical characteristics analysis, the HCC cases had corresponding clinical information and overall survival time was more than 30 days. The main characteristics of each dataset were listed in Additional file 13: Table S6 and the detail clinical traits of TCGA-LIHC cohort were provided in Additional file 14: Table S7.

### Preprocessing of training data

The GSE14520 dataset had been normalized by robust multi-array average (RMA) and GSE76427 dataset had been normalized by robust spline normalization (RSN, R package “lumi”) according to the metadata of these data sets from GEO database. To generate gene level summarization, we utilized an interquartile range (IQR) method. This allowed us to designate the probe set ID with the largest IQR of expression values out of all multiple probe set IDs as the representative of the gene. Missing expression values are imputed using nearest neighbor imputation (R package “impute”). ComBat (R package “sva”), an empirical Bayes method, was applied to achieve correct batch effect and cross-study normalization (Additional file 1: Fig. S1).

### Development of the model

Briefly, we extracted and included all pathway information from Kyoto Encyclopedia of Genes and Genomes [48–50], Pathway Interaction Database [51] and BioCarta Pathway Database [52]. Using R package “pathifier”, we transformed gene-level information into pathway-level information and gained a PDS matrix. And then we chose penalized regression with a global-tuning algorithm to get a model which can achieve a balance between explanatory ability and parsimony. For a detailed description of the method, please refer to Additional file 15: Doc. S1.

### Evaluation indicators

The AUROC, AUPRC, Brier score, accuracy (ACC), precision, recall, F1-score and MCC were utilized to assess the performance of this model. The ROC curve was a plot characterized by true positive rate [true positive/(true positive + false negative)] and false positive rate [false positive/(true negative + false positive)]. And the PRC curve was a plot characterized by precision and recall. F1-score could be seen as a harmonic average of the model precision and recall.

$${\text{ACC}} = \frac{{{\text{TP}} + {\text{TN}}}}{{{\text{TP}} + {\text{FP}} + {\text{TN}} + {\text{FN}}}}$$

$${\text{Precision}} = \frac{{{\text{TP}}}}{{{\text{TP}} + {\text{FP}}}}$$

$${\text{Recall}} = \frac{{{\text{TP}}}}{{{\text{TP}} + {\text{FN}}}}$$

$${\text{F}}1{\text{score}} = 2 \times \frac{{{\text{Precision}} \times {\text{Recall}}}}{{{\text{Precision}} + {\text{Recall}}}}$$

$${\text{MCC}} = \frac{{{\text{TP}} \times {\text{TN}} - {\text{FP}} \times {\text{FN}}}}{{\sqrt {\left( {{\text{FN}} + {\text{TN}}} \right)\left( {{\text{FP}} + {\text{TN}}} \right)\left( {{\text{TP}} + {\text{FN}}} \right)\left( {{\text{TP}} + {\text{FP}}} \right)} }}$$

where TP is positive examples correctly labeled as positive. FP is negative examples incorrectly labeled as positive. TN is negative examples correctly labeled as negative. FN is positive examples incorrectly labeled as negative.

### Survival analysis

The univariate significance of variables related to OS and RFS were determined by Kaplan–Meier method and log rank test. Influence of multiple variables on survival were tested through univariate and multivariate Cox regression analysis. The Cox proportional hazards parameters’ significance was detected by the Wald test and documented as the hazard ratio (HR), with 95% CI.

### Lasso Cox regression analysis

Lasso Cox regression analysis was performed to analyze samples and corresponding genes via glmnet function of R package “lars”, in which the parameters were alpha = 1 and nlambda = 100.

### SCNA data processing

Somatic copy-number alterations (SCNA) genomic features were defined as repetitive regions with copy-number changes determined by GISTIC2 [53]. The SCNA data processing method in the previous study [54] was used to determine SCNA features and binary states in each sample. The concrete methods were as follows: Peak regions from GISTIC results of all tumor types were extracted as SCNA features. As to peak regions of the same gene, only one peak region was retained. To determine the SCNA event, we use the discrete copy number calls provided by GISTIC: − 2, homozygous loss; − 1, heterozygous loss; 0, diploid; 1, single-copy gain; 2, high-level amplification or multiple-copy gain.

### CNV and SNV analysis

For CNV, the analysis was carried out by GISTIC2. The specific parameters used were as follows: − ta = 0.1, − armpeel = 1, brlen = 0.7, − cap = 1.5, − conf = 0.75, − td = 0.1, − genegistic = 1, − gcm = extreme, − js = 4, − maxseg = 2000, − qvt = 0.25, − rx = 0, − savegene = 1. And with respect to SNV, we applied default parameters of R package “maftool” to analyze the mutation of TCGA-LIHC dataset, whose statistical results were directly generated by oncplot function of package “maftool”.

### Correlation of gene signature with immune related pathways

Previous studies have constructed some gene sets as immune related pathways, including (1) immune checkpoint; (2) antigen processing machinery; (3) CD8 T-effector signature; (4) epithelialmesenchymal transition (EMT) markers including EMT1, EMT2 and EMT3; (5) Angiogenesis signature; (6) pan-fibroblast TGFb response signature (Pan-F-TBRS); (7) WNT targets; (8) DNA damage repair; (9) mismatch repair; (10) Nucleotide excision repair; (11) DNA replication; (12) Fanconi anemia; (13) Cell cycle; (14) Cell cycle regulators; (15) FGFR3 related genees; (16) Homologous recombination; (17) KEGG discovered histones [55–57]. We made a correlation analysis of the risk score generated from the gene signature and gene set variation analysis (GSVA) enrichment scores of these pathways.

### Statistical analysis

All statistical tests were performed by R software version 4.0.2. ANOVA or t.test was employed for differential analysis. And *P*-value < 0.05 was statistically significant.

## Supplementary Information

**Additional file 1: Fig. S1.** Preprocessing of training data.

**Additional file 2: Fig. S2.** Construction of hub gene network.

**Additional file 3: Fig. S3.** The Kaplan-Meier analysis results of OS and RFS about 33 characteristic genes. The left of each sub-figure is about OS and the right is about RFS.

**Additional file 4: Fig. S4.** Comparison of the expression of 12 genes of the risk signature in non-cancer and different stages of HCC tissues.

**Additional file 5: Fig. S5.** The Kaplan-Meier survival curve of the 12-gene signature for HCC patients with various clinicopathological characters in TCGA-LIHC cohort.

**Additional file 6: Fig. S6.** Analysis of enriched pathways and single nucleotide variations related to risk score or characteristic genes from the 12-gene signature.

**Additional file 7: Fig. S7.** The expression of 12 characteristic genes of the risk signature in HCC from HCCDB database. Diff: the number of differentially expressed datasets; Red/Blue for consensus up-regulated/down-regulated. Prognosis: the number of significant datasets by survival analysis; Red/Blue for Unfavorable/Favorable. HCC/All Tumor: Red/Blue for positive/negative fold change in log2 scale by comparing HCC with all tumors (TCGA data). HCC/All Adjacent: Red/Blue for positive/negative fold change in log2 scale by comparing HCC with all adjacent samples (TCGA data). HCC/Adjacent: Red/Blue for positive/negative fold change in log2 scale by comparing HCC with adjacent samples (HCCDB data). Liver Other Normal: Red/Blue for positive/negative fold change in log2 scale by comparing liver with normal tissues (GTEx&TCGA data).

**Additional file 8: Table S1.** Comparison of the diagnostic efficiency of different diagnostic models.

**Additional file 9: Table S2.** g:Profiler enrichment result.

**Additional file 10: Table S3.** Characteristic genes with significant prognostic value.

**Additional file 11: Table S4.** Multivariate Cox regression analysis of 12 genes for risk signature construction.

**Additional file 12: Table S5.** Univariate and multivariate Cox regression analysis of risk score and other clinical traits for OS in TCGA-LIHC cohort.

**Additional file 13: Table S6.** The datasets used in this study.

**Additional file 14: Table S7.** The clinical traits of TCGA-LIHC cohort.

**Additional file 15: Doc. S1.** Supplementary method for the development of the model.

## Data Availability

The datasets generated and analyzed during the current study are available in the [HCCDB] repository, [http://lifeome.net/database/hccdb/download.html]. Training and external validation sets, GSE14520 [https://www.ncbi.nlm.nih.gov/geo/query/acc.cgi?acc=GSE14520]. GSE76427 [https://www.ncbi.nlm.nih.gov/geo/query/acc.cgi?acc=GSE76427]. TCGA-LIHC [https://xenabrowser.net/datapages/].

## Abbreviations

AFP: Alpha-fetoprotein
AUPRC: Area under the precision-recall curve
AUROC: Area under the receiver operating characteristic curve
CI: Confidence interval
CNV: Copy number variation
CVM: Cross-study validation method
EPSGO: Efficient parameter selection via global optimization
GSVA: Gene set variation analysis
HCC: Hepatocellular carcinoma
HDLSS: High-dimension low sample size
HR: Hazard ratio
Lasso: Least absolute shrinkage and selection operator
MCC: Matthews correlation coefficient
OS: Overall survival
PDS: Pathway deregulation scores
PPI: Protein protein interaction
RFS: Relapse-free survival
RMA: Robust multi-array average
RSN: Robust spline normalization
SCNA: Somatic copy-number alterations
SNV: Single nucleotide variation
SVM: Support vector machine
