# Supplementary figures and images for: Machine-learning algorithms based on personalized pathways for a novel predictive model for the diagnosis of hepatocellular carcinoma
Source: BMC Bioinformatics. 2022 Jun 23;23:248. doi: 10.1186/s12859-022-04805-9 (PMC9219178; doi:10.1186/s12859-022-04805-9)

A

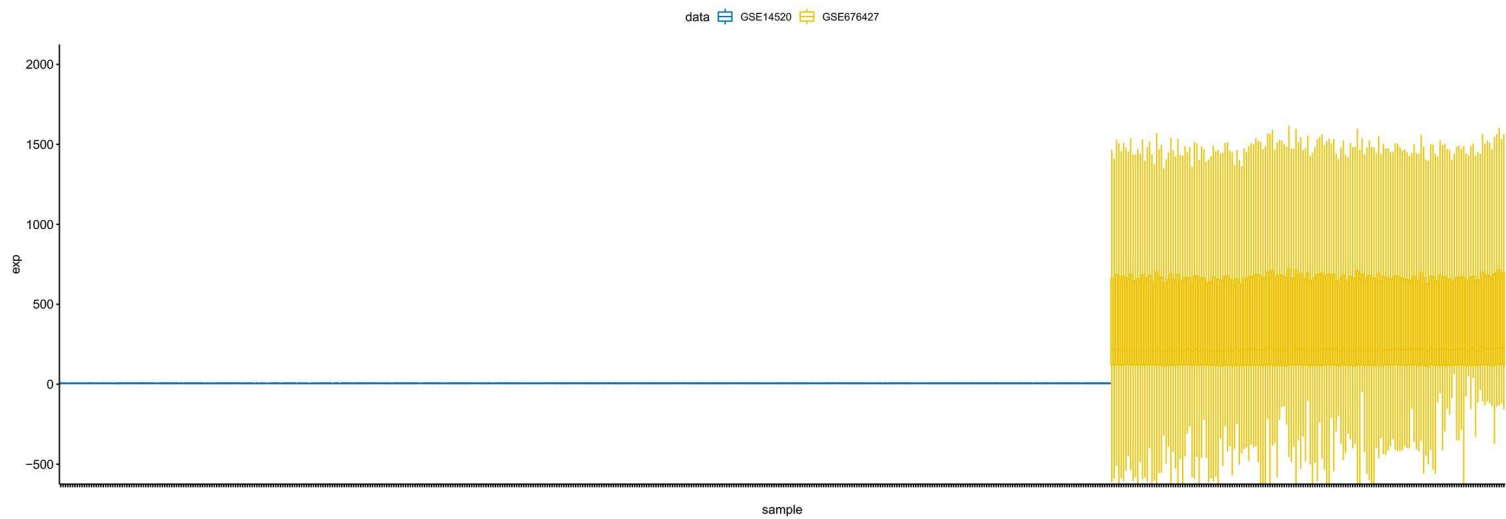

B

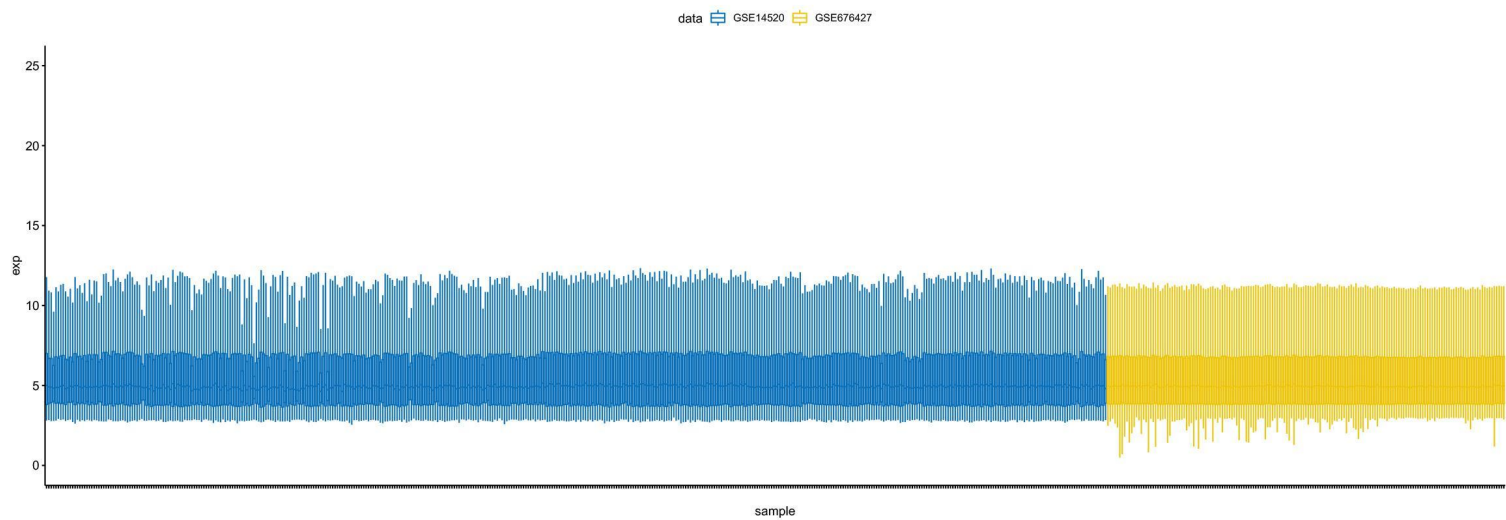

Additional file 1: Fig. S1 Preprocessing of training data.

Supplement: Supplementary file 1 — Additional file 1: Fig. S1. Preprocessing of training data. [file 12859_2022_4805_MOESM1_ESM.pdf]

A

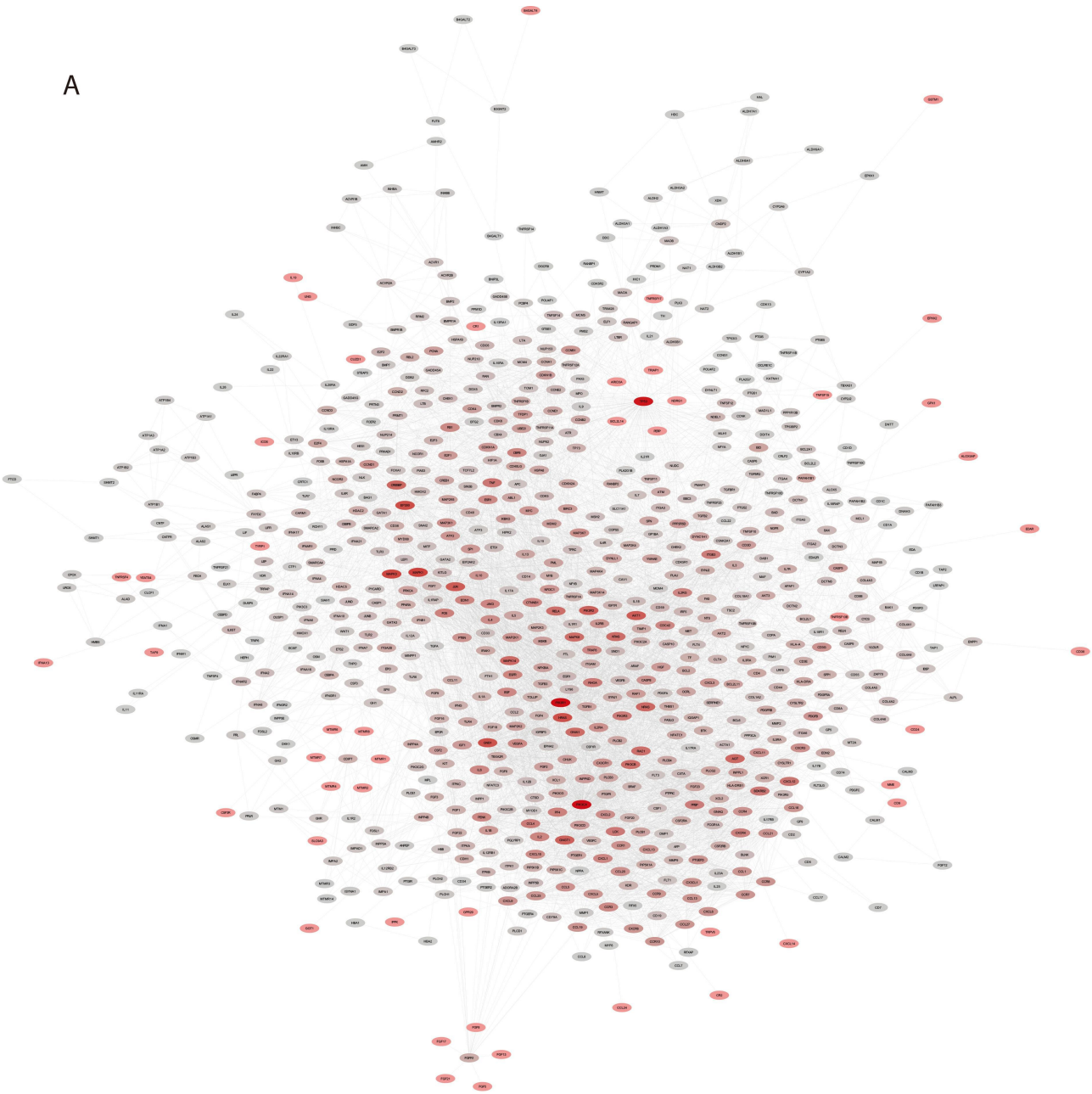

B

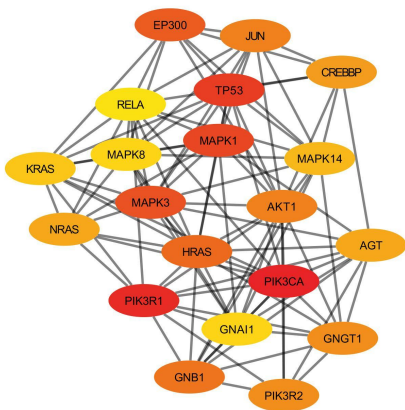

Additional file 2: Fig. S2 Construction of hub gene network.

Supplement: Supplementary file 2 — Additional file 2: Fig. S2. Construction of hub gene network. [file 12859_2022_4805_MOESM2_ESM.pdf]
