# Supplementary material for: Machine-learning algorithms based on personalized pathways for a novel predictive model for the diagnosis of hepatocellular carcinoma
Source: BMC Bioinformatics. 2022 Jun 23;23:248. doi: 10.1186/s12859-022-04805-9 (PMC9219178; doi:10.1186/s12859-022-04805-9)
Supplement: Supplementary file 3 — Additional file 3: Fig. S3. The Kaplan-Meier analysis results of OS and RFS about 33 characteristic genes. The left of each sub-figure is about OS and the right is about RFS. [file 12859_2022_4805_MOESM3_ESM.pdf]

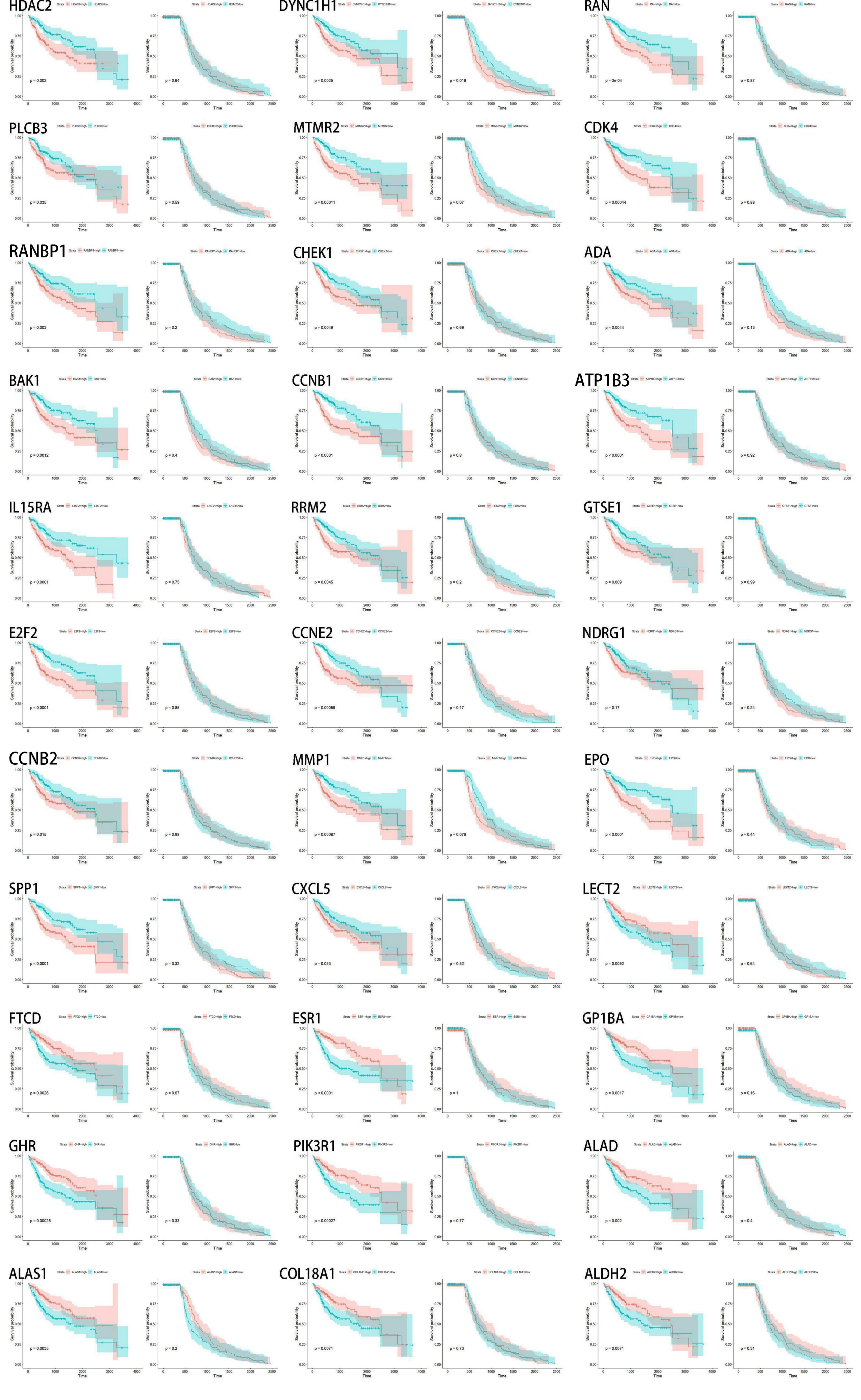

Additional file 3: Fig. S3 The Kaplan-Meier analysis results of OS and RFS about 33 characteristic genes. The left of each sub-figure is about OS and the right is about RFS.
