# Supplementary material for: Machine-learning algorithms based on personalized pathways for a novel predictive model for the diagnosis of hepatocellular carcinoma
Source: BMC Bioinformatics. 2022 Jun 23;23:248. doi: 10.1186/s12859-022-04805-9 (PMC9219178; doi:10.1186/s12859-022-04805-9)
Supplement: Supplementary file 4 — Additional file 4: Fig. S4. Comparison of the expression of 12 genes of the risk signature in non-cancer and different stages of HCC tissues. [file 12859_2022_4805_MOESM4_ESM.pdf]

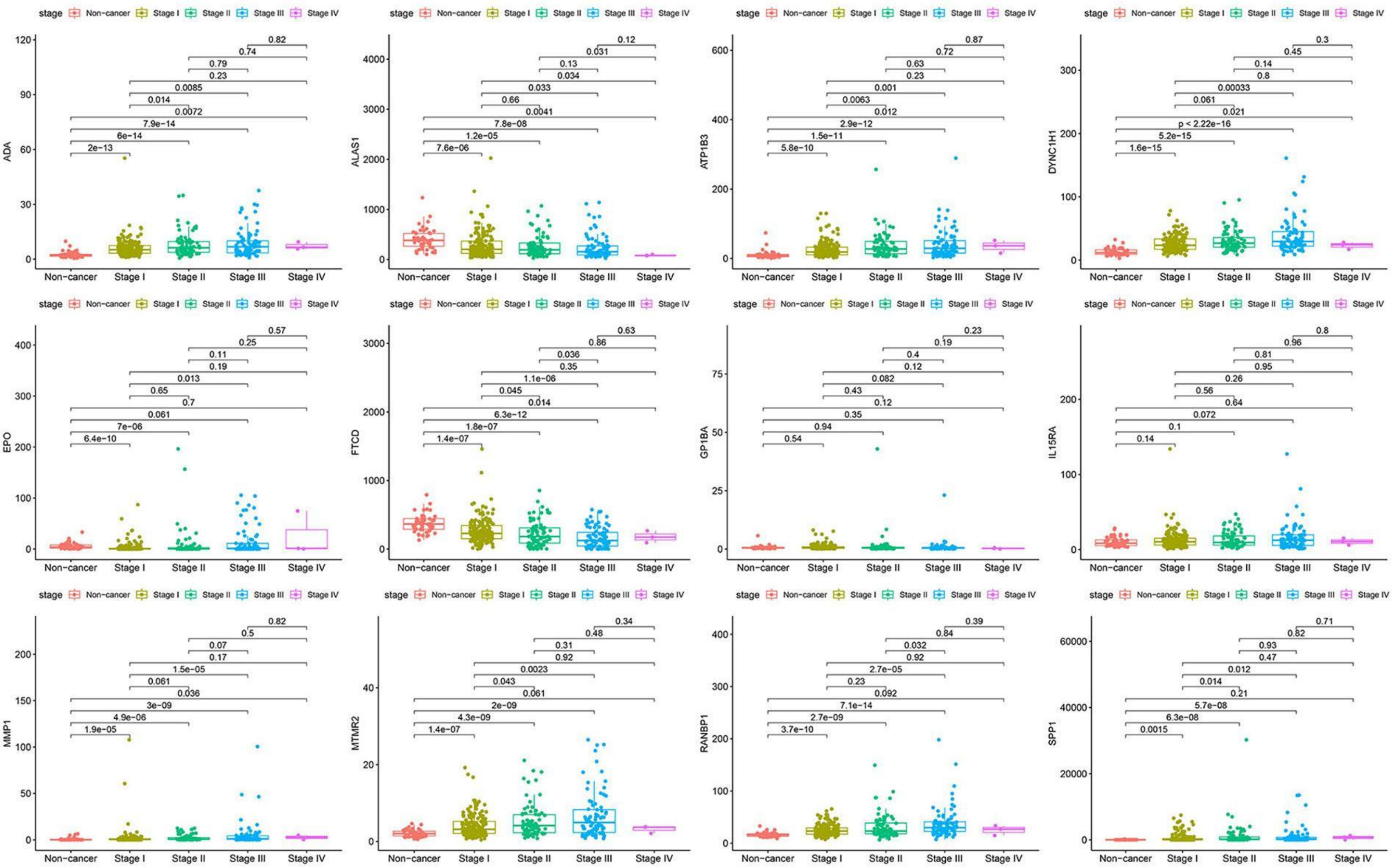

Additional file 4: Fig. S4 Comparison of the expression of 12 genes of the risk signature in non-cancer and different stages of HCC tissues.
