# Supplementary material for: Machine-learning algorithms based on personalized pathways for a novel predictive model for the diagnosis of hepatocellular carcinoma
Source: BMC Bioinformatics. 2022 Jun 23;23:248. doi: 10.1186/s12859-022-04805-9 (PMC9219178; doi:10.1186/s12859-022-04805-9)
Supplement: Supplementary file 5 — Additional file 5: Fig. S5. The Kaplan-Meier survival curve of the 12-gene signature for HCC patients with various clinicopathological characters in TCGA-LIHC cohort. [file 12859_2022_4805_MOESM5_ESM.pdf]

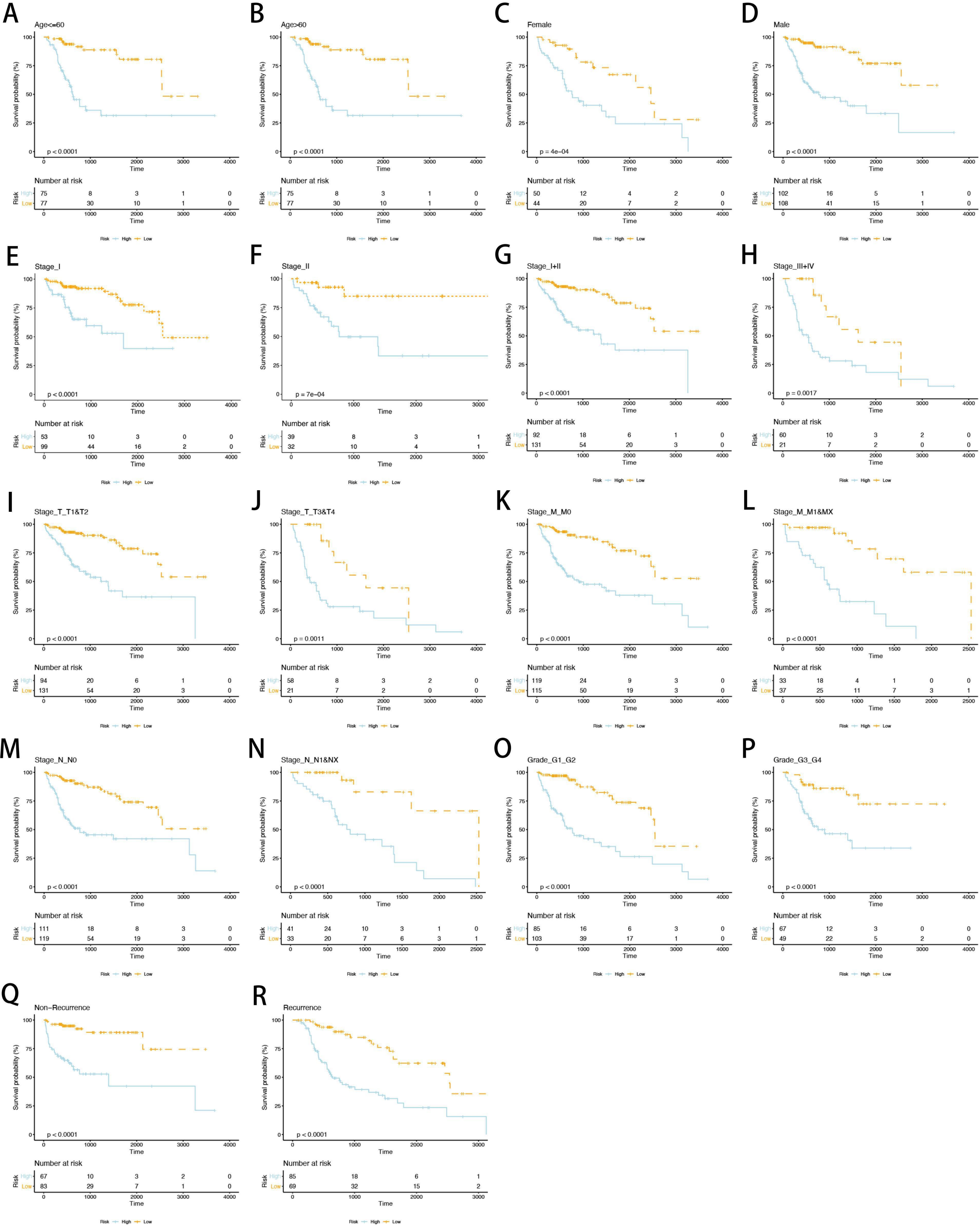

Additional file 5: Fig. S5 The Kaplan-Meier survival curve of the 12-gene signature for HCC patients with various clinicopathological characters in TCGA-LIHC cohort.
