# Supplementary material for: Machine-learning algorithms based on personalized pathways for a novel predictive model for the diagnosis of hepatocellular carcinoma
Source: BMC Bioinformatics. 2022 Jun 23;23:248. doi: 10.1186/s12859-022-04805-9 (PMC9219178; doi:10.1186/s12859-022-04805-9)
Supplement: Supplementary file 6 — Additional file 6: Fig. S6. Analysis of enriched pathways and single nucleotide variations related to risk score or characteristic genes from the 12-gene signature. [file 12859_2022_4805_MOESM6_ESM.pdf]

A

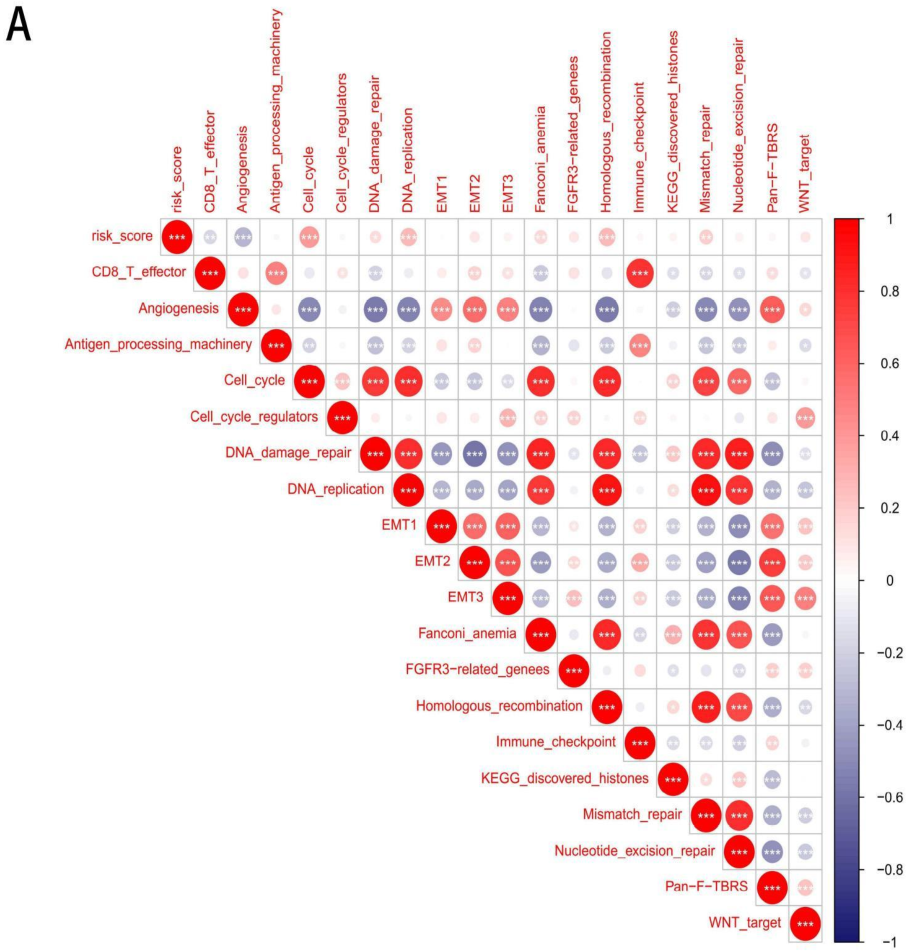

B

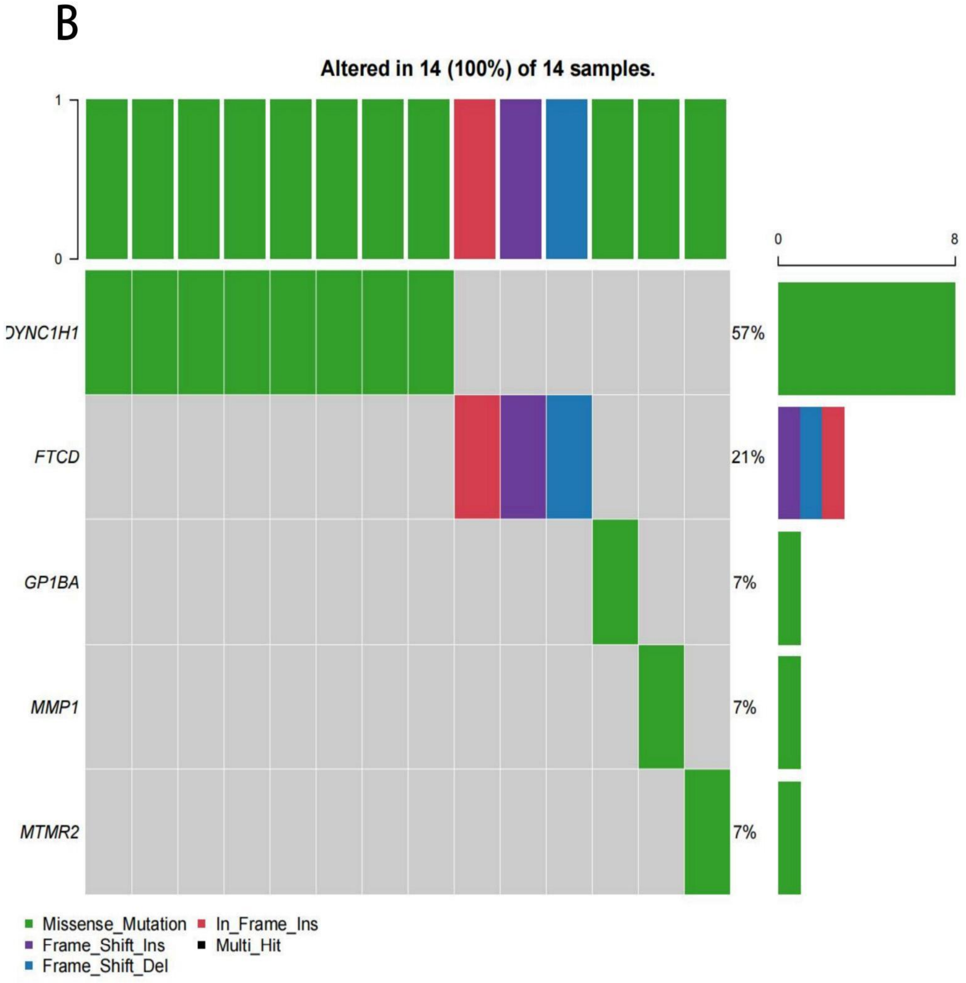

Additional file 6: Fig. S6 Analysis of enriched pathways and single nucleotide variations related to risk score or characteristic genes from the 12-gene signature.
