# Supplementary material for: Machine-learning algorithms based on personalized pathways for a novel predictive model for the diagnosis of hepatocellular carcinoma
Source: BMC Bioinformatics. 2022 Jun 23;23:248. doi: 10.1186/s12859-022-04805-9 (PMC9219178; doi:10.1186/s12859-022-04805-9)
Supplement: Supplementary file 7 — Additional file 7: Fig. S7. The expression of 12 characteristic genes of the risk signature in HCC from HCCDB database. Diff: the number of differentially expressed datasets; Red/Blue for consensus up-regulated/down-regulated. Prognosis: the number of significant datasets by survival analysis; Red/Blue for Unfavorable/Favorable. HCC/All Tumor: Red/Blue for positive/negative fold change in log2 scale by comparing HCC with all tumors (TCGA data). HCC/All Adjacent: Red/Blue for positive/negative fold change in log2 scale by comparing HCC with all adjacent samples (TCGA data). HCC/Adjacent: Red/Blue for positive/negative fold change in log2 scale by comparing HCC with adjacent samples (HCCDB data). Liver Other Normal: Red/Blue for positive/negative fold change in log2 scale by comparing liver with normal tissues (GTEx&TCGA data). [file 12859_2022_4805_MOESM7_ESM.pdf]

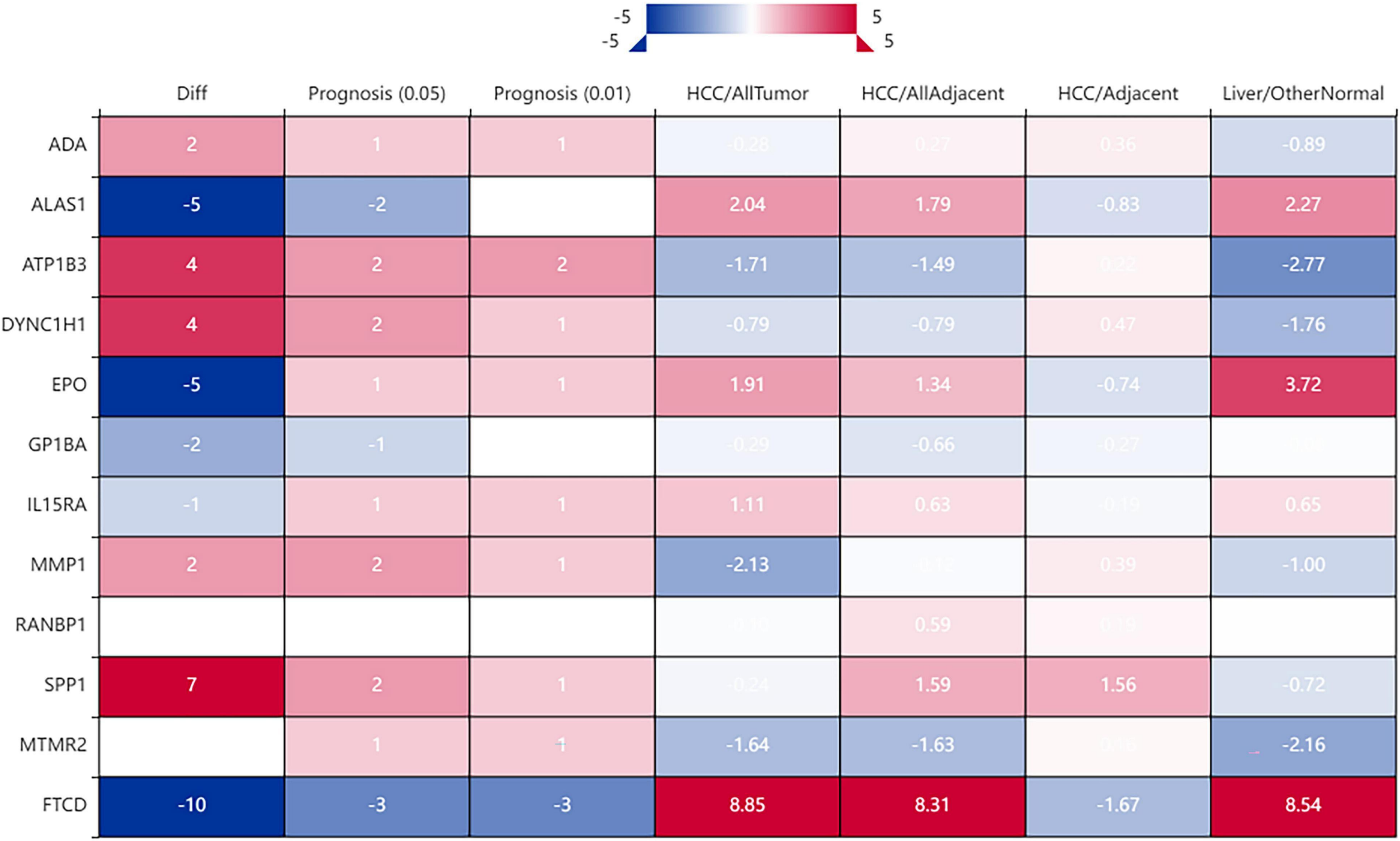

Additional file 7: Fig. S7 The expression of 12 characteristic genes of the risk signature in HCC from HCCDB database. Diff: the number of differentially expressed datasets; Red/Blue for consensus up-regulated/down-regulated. Prognosis: the number of significant datasets by survival analysis; Red/Blue for UNFavorable/Favorable. HCC/AllTumor: Red/Blue for positive/negative fold change in log2 scale by comparing HCC with all tumors (TCGA data). HCC/AllAdjacent: Red/Blue for positive/negative fold change in log2 scale by comparing HCC with all adjacent samples (TCGA data). HCC/Adjacent: Red/ Blue for positive/negative fold change in log2 scale by comparing HCC with adjacent samples (HCCDB data). LiverOtherNorm al: Red/Blue for positive/negative fold change in log2 scale by comparing liver with normal tissues (GTEx&TCGA data).
