# Supplementary material for: Machine-learning algorithms based on personalized pathways for a novel predictive model for the diagnosis of hepatocellular carcinoma
Source: BMC Bioinformatics. 2022 Jun 23;23:248. doi: 10.1186/s12859-022-04805-9 (PMC9219178; doi:10.1186/s12859-022-04805-9)
Supplement: Supplementary file 15 — Additional file 15: Doc. S1. Supplementary method for the development of the model. [file 12859_2022_4805_MOESM15_ESM.pdf]

Additional file 15: Doc. S1 Supplementary method for the development of the model.

The method for the development of the model was based on a previous report[1]. Briefly, Hastie and Stuetzle's principal curves algorithm [2] and Pathifier algorithm [3] were utilized to calculate a principal curve and deregulation score for each pathway, respectively. A one-dimensional principal curve was yielded by analyzing data points from the 'middle' of data cloud in the high dimensional space, which well represent the average value of the data density. The principal curve *f* in *n*−dimensional space is a vector *f* (*v*) of *n* functions of a single parameter variable *v*. Based on a finite *n*−dimensional random vector $X = (X_1, X_2, \dots X_n)$,the projection index is defined as: $v_f(x) = \sup_v \{v: ||x - f(v)|| = \inf_\mu ||x - f(\mu)||\}$ and the condition for self-consistency is simply $f(v) = E(X \mid v_f(X) = v)$. Every sample*(i)*'s pathway (*P*) deregulation score $D_P(i)$ was calculated by the distance along the curve between principal curve f_i and a reference point *c* (the centroid of the control samples). The pathway information was extracted from Kyoto Encyclopedia of Genes and Genomes [4], Pathway Interaction Database [5] and BioCarta Pathway Database [6]. Then the model was constructed through regularization techniques by using R package glmnet [7], which contains elastic net model that linearly combines the penalties of the lasso and ridge regression methods. Two hyperparameters (α and λ) needed be fine-tuned to obtain a suitable elastic-net penalty function. The trade-off between the ridge and lasso penalties was dominated by α, while the total amount of penalization was controlled by λ. Furtherly, the function in the R package C060 were used to make a multi-study-derived classifier [8]. Using the leave-one-out cross-validation (LOOCV) procedure, the average standard error and optimal value of the regularization parameter with minimum deviance were determined. Then the parameters were further optimized by using the efficient parameter selection via global optimization (EPSGO) algorithm [8], which is robust against local minima and has extremely high computational efficiency. Finally, the optimal parameter values (α= 0.85372, λ= 0.004230762, deviance= 0.03503) were obtained for variable selection in this study.

Supplementary References

1. Park KS, Kim SH, Oh JH & Kim SY (2021) Highly accurate diagnosis of papillary thyroid carcinomas based on personalized pathways coupled with machine learning. Briefings in bioinformatics 22, doi: 10.1093/bib/bbaa336.
2. Hastie T, Stuetzle W. Principal Curves. J. Am. Stat. Assoc. 1989; 84:502–516
3. Drier Y, Sheffer M, Domany E. Pathway-based personalized analysis of cancer. Proc Natl Acad Sci USA 2013; 110:6388–6393
4. Kanehisa M & Goto S (2000) KEGG: kyoto encyclopedia of genes and genomes. Nucleic acids research 28, 27-30, doi: 10.1093/nar/28.1.27.
5. Schaefer CF, Anthony K, Krupa S, Buchoff J, Day M, Hannay T & Buetow KH (2009) PID: the Pathway Interaction Database. Nucleic acids research 37, D674-679, doi: 10.1093/nar/gkn653.
6. D N (2001) BioCarta. Biotech Software & Internet Report 2. Open Access Library 117–20.
7. Friedman J, Hastie T, Tibshirani R. Regularization Paths for Generalized Linear Models via Coordinate Descent. J. Stat. Softw. 2010; 33:1–22
8. Sill M, Hielscher T, Becker N, et al. c060 : Extended Inference with Lasso and Elastic-Net Regularized Cox and Generalized Linear Models. J. Stat. Softw. 2014; 62:1–22
